# Book Notices and Reviews

**Published:** 1865-09

**Authors:** 


### BOOK NOTICES AND REVIEWS.

*A Treatise on Military Surgery and Hygiene.* By Frank Hastings Hamilton, M. D., late Lieutenant-Colonel, Medical Inspector U. S. A.; Professor of Military Surgery and Hygiene, and of Fractures and Dislocations, in Bellevue Medical College, Surgeon to Bellevue Hospital, Author of a Treatise on "Fractures and Dislocations," and of a "Practical Treatise on Military Surgery." Illustrated with 127 Engravings. New York: Bailliere Brothers, 520 Broadway. 1865.

A suitable notice of this work has been too long delayed, and we now introduce it to the readers of the JOURNAL, without attempting a complete analysis, feeling assured that all who are in any way interested in this important branch of Surgery, will early purchase and peruse the work for themselves.

It is a new and original work by one of the leading Surgeons of the country, and it is enriched by the experience of four years in the camp and field of actual war. The opportunity thus offered to observe the progress and improvements in the art of Military Surgery has not been limited nor unimproved by the author, which give to the teachings of the work a value and authority which no merely theoretical dissertation could possibly impart.

The magnitude of the conflict through which we have just passed, is conceded to have been unequaled in any former time, and nothing was more likely than that such a contest would develop the inventive genius of the nation to the utmost in improving and perfecting the weapons of the belligerents, and accordingly we find that the art of war has reached a point of perfection unapproached hitherto. Great as have been the demonstrations of genius on the part of the combatants, however, improvement has by no means been limited to them, for the effectiveness of the army, in its most brilliant campaigns, is undeniably in a great measure due to the skill and judgment of the Surgical corps in enforcing a more general observance of the laws of hygiene, and in that conservative Surgery which seeks to preserve the *limbs* as well as the lives of our soldiers.

We subjoin the table of contents, which, with the known ability of the author, will lead to a just appreciation of the value of the work:—Introduction—Examination of Recruits—General Hygiene of Troops—Bivouac, Accommodation of Troops in Tents, Barracks, Billets, Huts, etc.—Hospitals—Preparations for the Field—Hygienic Management of Troops upon the March—Conveyance of Sick and Wounded Soldiers—Gunshot Wounds—Gunshot Injuries of the Head—Gunshot Injuries of the Face and Neck—Gunshot Wounds of the Thorax—Punctured and Incised Wounds of the Thorax—Gunshot Wounds of the Abdomen—Punctured and Incised Wounds of the Abdomen—Gunshot Wounds of the Male Organs of Generation—Gunshot Fractures—Amputations—Exsections—Arrow Wounds—Traumatic Gangrene—Hospital Gangrene—Dry Gangrene—Tetanus—Scorbutus—On the Employment of Anæsthetics in Major Amputations and in other Severe Surgical Operations, after Gunshot Injuries—Appendix.

---

*A System of Surgery; Pathological, Diagnostic, Therapeutic, and Operative.* By Samuel D. Gross, M. D. Illustrated by over thirteen hundred Engravings. Third edition, much enlarged and revised. In two volumes. Published by Blanchard and Lea, Philadelphia.

The first two editions of Prof. Gross's System of Surgery are so familiar to the profession, and so highly prized, that it would be idle for us to speak in praise of this work. It is bound in calf, and the two volumes contain above two thousand pages. We cannot give an idea of the changes introduced into the last edition more briefly than to quote from the author's preface: "Upon the edition now issued upwards of two years and a half of arduous labor have been expended.

Every chapter has been thoroughly revised; the text has been augmented by an amount of matter nearly equal to two hundred pages; and a considerable number of wood-cuts, nearly all prepared for the purpose, have been introduced. Many portions have been entirely re-written, and every effort has been made to condense the language; while an enlargement in the form of the work has prevented an increase in the number of pages. The general arrangement is the same as in the previous imprints; and the additions, for the most part, widely scattered through the text, are essentially of a practical character."

---

*The Essentials of Materia Medica and Therapeutics.* By Alfred Baring Garrod, M. D., F.R.S., Fellow of the Royal College of Physicians, Professor of Materia Medica and Therapeutics at King's College, London; Physician to King's College Hospital, and Examiner in Materia Medica in the University of London. Published by William Wood & Co., 61 Walker street, N. Y. 1865.

This volume of 400 pages treats only of Materia Medica, it being the purpose and hope of the author "in a few months, to bring before the profession a separate volume devoted exclusively to the value of medicines in the treatment of disease, and embracing the whole subject of Therapeutics." The work is prepared with much ability, the natural history of medicines, their chemical relations, most common adulterations and doses, are given methodically, clearly and concisely, while even their best established therapeutic uses are not omitted, though condensed in a brief space. It is a very valuable addition to our medical literature, and we shall look with interest for the publication of its "companion work" on Therapeutics. Each book is to be complete in itself. The price of the present volume is $4.

---

We take pleasure in calling the attention of our readers to the "Price List" of S. C. Griggs & Co., contained in this number. This enterprising house promptly add to their large stock all recent medical publications.

GLAUCOMA: ITS SYMPTOMS, DIAGNOSIS AND TREATMENT, by PETER DIRCK KEYSER, M. D.; *Philadelphia, Lindsay & Blakiston*, 1864. *For Sale at Griggs & Co., Lake st., Chicago.*

Our readers who have not studied the subject of Glaucoma, as discussed in the original papers of Graefe, and other eminent observers, will find much profit in the careful perusal of this monograph. We do not recommand the work on the ground that Glaucoma is a common disease, and the subject therefore of great practical importance to the general physician. At the West the disease is undoubtedly very rare, on account perhaps of the small proportion in our population of aged persons, who are specially liable to it.

The use of the ophthalmoscope and Graefe's and Hancock's brilliant discoveries of iridectomy and section of the ciliary muscle, as curative operations for the disease, have stimulated investigation and extended our knowledge of this subject.

As an exposition of many well established facts regarding certain diseases of the intraocular tissues, we would commend the monograph of Dr. Keyser. It is stated in the preface that the work is founded on the facts observed in the clinics of Graefe at Berlin. We believe the *Boston Medical and Surgical Journal* was the first to call attention of the profession to the fact that the pamphlet is a literal reprint of the lectures of J. S. Wells, on Glaucoma, published last year in London. The work is yet worthy of perusal, although we think very much more could be said in favor of Hancock's operation of Paracentesis oculi with division of the ciliary muscle, than appears in the work before us.

The typography is worthy of notice. The peculiar tint of the paper will undoubtedly be found agreeable to the eye of the reader. Probably all books, if made of yellow-tinted paper would be read with less fatigue by the majority of persons than as now printed upon perfectly white paper.

